# Numerical Study on Stochastic Diabetes Mellitus Model with Additive Noise

**DOI:** 10.1155/2019/5409180

**Published:** 2019-06-10

**Authors:** Zhifang Zhang, Qingyi Zhan, Xiangdong Xie

**Affiliations:** ^1^Fujian Center for Disease Control and Prevention, Fuzhou, Fujian 350002, China; ^2^College of Computer and Information Science, Fujian Agriculture and Forestry University, Fuzhou 350002, China; ^3^Department of Mathematics, Ningde Normal University, Ningde, Fujian 352100, China

## Abstract

This article focuses on the numerical analysis and simulation of the stochastic diabetes mellitus model with additive noise. The existence and uniqueness theorem of the solution under some appropriate assumptions is established. And, the mean square stability and convergence of numerical solutions are proposed, too. The practical use of these theorems is demonstrated in the numerical computations of the stochastic diabetes mellitus model and the value for the forecast of the tendency of diabetes mellitus in a given time.

## 1. Introduction

At present, with the development of the society and the increment of the economy, diabetes mellitus is becoming more and more popular in the world. In fact, diabetes mellitus is the name given to a group of different conditions in which there is not the right amount of insulin to stabilize the amount of sugar in the body. As we know, there exist two forms of diabetes mellitus. Type I diabetes mellitus, which depends on insulin, most often occurs in young people, while type II diabetes mellitus, which does not depend on insulin, usually develops in the aged.

It is well known that there are many finished works which utilize two methods to investigate the diabetes mellitus in the view of mathematical model. One pays attention to microscopic action of the nosogenesis of diabetes mellitus and forms many mathematical models such as ordinary differential equations and partial differential equation (refer [1] and the references therein). Another takes account of the macroscopic case of the size of population of diabetes mellitus in a given time. This leads to insight into the control of diabetes mellitus, and with the current alarming increase in the incidence of the disease of a given considered region, this area has gained increased interest and importance.

However, due to many uncertainties or random influences, where these uncertainties come from the influence of diet, physical activity level, and the age dynamical distribution of population, noises should be taken into account. The existing deterministic mathematical models of diabetes mellitus [2] need to be revised so that it can simulate the fact more really. Therefore, we expand it to the case of stochastic differential equations (SDEs), whose applications describe many natural phenomena in meteorology, biology, and so on [3, 4]. As far as we know, till now, there has been little investigation of the diabetes mellitus mathematical model in the view of SDEs in the literature. Stochastic numerical analysis is still an interesting method of studying epidemic disease tendency of diabetes mellitus.

The main motivations of this work are twofold. On one side of the coin, the classical results about the deterministic mathematical model of diabetes mellitus are the base of this research. A variety of mathematical models have been used for different aspects of diabetes mellitus, and many important results, which can reveal the facts of diabetes mellitus, are obtained (refer [2, 5–9] and the references therein). On the other hand, it has been attracted by some random phenomena which often appear in the population dynamics of diabetes mellitus. These need numerical simulations to direct the control policy of diabetes mellitus. Furthermore, it is the fact that there exists our earlier work [10, 11] on stability analysis and numerical simulation of SDEs. For example, there are some work on numerical analysis of SDEs [11–15] and numerical simulation of SDEs [10]. These carry out the foundation of numerical analysis [16].

In this work, we first prove the existence and uniqueness theorem of the solution of the stochastic diabetes mellitus model under some assumptions. Then, the mean square stability and convergence are proposed. And, numerical examples are shown to illustrate the possibility of the stochastic mathematical model of diabetes mellitus and the value in the forecast of the tendency of diabetes mellitus in a given time. These results show that, under some appropriate conditions, SDEs can simulate the epidemic disease tendency of diabetes mellitus more accurately, whose value can be well estimated by the numerical approximative solution.

A more detailed outline of this paper is as follows.  Section 2 shows some relevant concepts and norms which will be utilized later.  Section 3 is devoted to the theoretical analysis of the stochastic diabetes mellitus model, that is, the existence of the solution, mean square stability, and convergence.  Section 4 presents numerical experiments of the stochastic diabetes mellitus model in some given areas. Illustrative numerical results for the main theorem are included.  Section 5 is addressed to the conclusions of this article.

## 2. Preliminaries

### 2.1. Generated Differential Equation Model

Here, we consider a stochastic model which can describe the dynamic behaviour of diabetes mellitus.

Let (*Ω*, *ℱ*, *ℙ*) be a canonical Wiener space, {*ℱ*_*t*_}_*t*∈*ℝ* ^+^_ be its natural normal filtration, and *W*(*t*)(*t* ∈ *ℝ* ^+^) be a standard one-dimensional Brownian motion defined on the space (*Ω*, *ℱ*, *ℙ*). We assume that *Ω*≔{*ω* ∈ *C*(*ℝ* ^+^, *ℝ*) : *ω*(0)=0} endowed with the compact-open topology. In the realization, *W*_*t*_(*ω*)≔*ω*(*t*), where *ω*(·) ∈ *Ω*, which means that the elements of *Ω* can be identified with the paths of the Wiener process. Based on the conclusions of deterministic ordinary differential equations about diabetes mellitus in [1, 2], we consider a class of Itô SDEs in the form of

(1) $$\begin{array}{c} \left\{\begin{array}{c} dC\left(t\right)=\left(-\left(\lambda \left(t\right)+\theta \left(t\right)\right)C\left(t\right)+\lambda \left(t\right)N\left(t\right)\right)dt+g_{1}\left(t\right)dW_{t},\\ dN\left(t\right)=\left(I\left(t\right)-\left(\nu \left(t\right)+\delta \left(t\right)\right)C\left(t\right)-\mu \left(t\right)N\left(t\right)\right)dt+g_{2}\left(t\right)dW_{t}, \end{array}\right. \end{array}$$

where *t* ∈ *ℝ* ^+^; the quantity of diabetes mellitus which has complications in a special research region at time *t* is written as *C*(*t*)(*C*(*t*) ∈ *ℝ* ^+^), and *C*(0)=*C*_0_; *N*(*t*) is on behalf of the scale of the population which has diabetes mellitus in a special research region at time *t*, namely, *N*(*t*)=*C*(*t*)+*D*(*t*). Here, *D*(*t*)(*D*(*t*) ∈ *ℝ* ^+^) presents the quantity of diabetes mellitus which has no complications in a special research region at time *t*; the morbidity of diabetes mellitus in a special research region at time *t* is represented as *I*(*t*); *μ*(*t*) stands for the mortality rate, the chance of a diabetes mellitus person who is developing a complication is written as *λ*(*t*), the proportion whose complications are mended is shown as *γ*(*t*), the parameter *ν*(*t*) presents the rate at which diabetic patients with complication become severely disable, the parameter *δ*(*t*) shows the mortality rate due to complications, *θ*(*t*)≔*γ*(*t*)+*μ*(*t*)+*ν*(*t*)+*δ*(*t*) denotes the sum of the above parameters, and *g*_1_(*t*) and *g*_2_(*t*) are functions with respect to *t*, which denote the uncertain influences [2].

### 2.2. Basic Notations and Assumptions

We make use of the following notations which is similar to [11]:

- Let *L* ^2^(*Ω*, *ℙ*) be the space of all square-integrable random variables *x* : *Ω*⟶*ℝ* ^*d*^.
- The norm of a random variable *x*=(*x*_1_, *x*_2_,…, *x*_*d*_) ∈ *L* ^2^(*Ω*, *ℙ*) is defined as

(2) $$\begin{array}{c} {\left\|x\right\|}_{2}={\left[\int_{\Omega }\left[{\left|x_{1}\left(\omega \right)\right|}^{2}+{\left|x_{2}\left(\omega \right)\right|}^{2}+,\ldots ,+\;{\left|x_{d}\left(\omega \right)\right|}^{2}d\mathbb{P}\left(\omega \right)\right]\right]}^{1/2}<\infty . \end{array}$$

(iii) The norm of a stochastic process *x*(*t*, *ω*) is defined as ||*x*(*t*, *ω*)||_2_=sup_*t*∈*ℝ* ^+^_‖*x*_*t*_(*ω*)‖_2_ < *∞*, where *x*_*t*_(*ω*) ∈ *L* ^2^(*Ω*, *ℙ*) and *t* ∈ *ℝ* ^+^.
(iv) We define the norm of random matrix *A*_*L* ^2^(*Ω*, *ℙ*)_=[*𝔼*(|*A*|^2^)]^1/2^, where *A* is a random matrix and |·| is the operator norm.
(v) Unless otherwise stated, the norms ‖·‖_2_ and ‖·‖_*L* ^2^(*Ω*, *ℙ*)_ are usually denoted as ‖·‖ in sequels.

In this paper, we also make the following assumptions which are used for the theoretical analysis [11].

**Hypothesis 1 .**

(i) The initial values *C*_0_ and *D*_0_ are bounded; that is,
(3) $$\begin{array}{c} \max\left\{\left\|C_{0}\right\|,\left\|D_{0}\right\|\right\}\leq K_{1}, \end{array}$$
for *K*_1_ > 0
(ii) Assume that the function *λ* : *ℝ*⟶*ℝ* is continuous, measurable function and the function *θ* : *ℝ*⟶*ℝ* is continuous, too
(iii) The functions *λ* and *θ* are globally bounded with respect to *t*. That is, there exists a positive constant *J* such that

(4) $$\begin{array}{c} \underset{t}{\max}\left\{\left|\theta \left(t\right)\right|,\left|\lambda \left(t\right)\right|\right\}\leq J, \end{array}$$

  holds for *t* ∈ *ℝ* ^+^
(iv) The functions *g*_1_(*t*) and *g*_2_(*t*) are globally bounded. That is, there exists a constant *K*_2_ > 0 such that

(5) $$\begin{array}{c} \underset{t}{\max}\left\{\left|g_{1}\left(t\right)\right|,\left|g_{2}\left(t\right)\right|\right\}\leq K_{2}, \end{array}$$

  holds for *t* ∈ *ℝ* ^+^

### 2.3. Equivalent Form

SDE (1) can be rewritten in the matrix-vector form as follows:

(6) $$\begin{array}{c} dX=b\left(X,t\right)dt+B\left(X,t\right)dW_{t}, \end{array}$$

where

(7) $$\begin{array}{c} X\left(t\right)=\left(\begin{array}{c} N\left(t\right) \end{array}\right),\\ B\left(X,t\right)=\left(\begin{array}{c} g_{2}\left(t\right) \end{array}\right), \end{array}$$

(8) $$\begin{array}{c} b\left(X,t\right)=\left(\begin{array}{c} I\left(t\right)-\left(\nu \left(t\right)+\delta \left(t\right)\right)C\left(t\right)-\mu \left(t\right)N\left(t\right) \end{array}\right). \end{array}$$

We define

(9) $$\begin{array}{c} \theta :\left(-\infty ,+\infty \right)\times \Omega ⟶\Omega ,\theta_{t}\omega \left(s\right)=\omega \left(t+s\right)-\omega \left(t\right). \end{array}$$

and Δ≔{(*s*, *t*) ∈ *ℝ* ^2^, *s* ≤ *t*}. By the conclusions in [3], SDE (1) generates a stochastic flow *φ* : Δ × *ℝ* ^2^ × *Ω*⟶*ℝ* ^2^ when the solution of SDE (1) exists uniquely, which is usually written as *φ*(*s*, *t*, *x*_0_, *ω*)≔*φ*(*s*, *t*, *ω*)*x*_0_ on the metric dynamical systems (*Ω*, *ℱ*, *ℙ*, *θ*_*t*_). The stochastic flow *φ* is given by

(10) $$\begin{array}{c} \varphi \left(s,t,\omega \right)X_{0}=X_{0}+\int_{s}^{t}b\left(\varphi \left(s,r,\omega \right)X_{0},r\right)dr+\int_{s}^{t}B\left(\varphi \left(s,r,\omega \right)X_{0},r\right)dW_{r},\;t\geq s. \end{array}$$

## 3. Theoretical Results

### 3.1. Existence of Equation (1)'s Solutions

The following result guarantees the existence of solutions for SDEs and is a direct consequence of Theorem 3.2.4 in [17].

**Lemma 1 .**

Suppose that *b* : *ℝ* ^2^ × [0, *T*]⟶*ℝ* ^2^ and *B* : *ℝ* ^2^ × [0, *T*]⟶*𝕄* ^2^ are continuous and satisfy the following conditions for some constant *L*:

-
$\left\|b\left(X,t\right)-b\left(\hat{X},t\right)\right\|\leq L\left\|X-\hat{X}\right\|,\;\;\left\|B\left(X,t\right)-B\left(\hat{X},t\right)\right\|\leq L\left\|X-\hat{X}\right\|$ for all 0 ≤ *t* ≤ *T* and $X,\hat{X}\in {\mathbb{R}}^{2}$

- ‖*b*(*X*, *t*)‖ ≤ *L*(1+‖*X*‖), ‖*B*(*X*, *t*)‖ ≤ *L*(1+‖*X*‖) for all 0 ≤ *t* ≤ *T* and *X* ∈ *ℝ* ^2^
- Let *X*_0_ be any *ℝ* ^2^-valued random variable such that *𝔼*(|*X*_0_|^2^) < *∞* and *X*_0_ is independent of *ℱ*_0_

Then, there exists a unique solution *X* ∈ *L* ^2^(*Ω*, *ℙ*) of the stochastic differential equation:

(11) $$\begin{array}{c} \left\{\begin{array}{c} dX=b\left(X,t\right)dt+B\left(X,t\right)dW_{t},\;\left(0\leq t\leq T\right),\\ X\left(0\right)=X_{0}. \end{array}\right. \end{array}$$

By the conclusions of Lemmas 3.1, we obtain the following theorem:

**Theorem 1 .**

Suppose that SDE (1) satisfies Hypothesis 2.1 and the initial conditions are given in Section 2.1.

Then, SDE (1) has a uniqueness solution *X*(*t*)=(*C*(*t*), *N*(*t*)) for all 0 ≤ *t* ≤ *T*.

**Proof**

In order to utilize Lemma 3.1 to this problem, we only need to check that the conditions of this theorem satisfy its three hypotheses.

First and foremost, Hypothesis (iii) obviously holds.

Secondly, by the assumptions of SDE (1), we obtain that

(12) $$\begin{array}{c} \left\|b\left(X,t\right)-b\left(\hat{X},t\right)\right\|=\left\|\begin{array}{c} -\left(\lambda \left(t\right)+\theta \left(t\right)\right)\left(C\left(t\right)-\hat{C}\left(t\right)\right)+\lambda \left(t\right)\left(N\left(t\right)-\hat{N}\left(t\right)\right)\\ -\left(\nu \left(t\right)+\delta \left(t\right)\right)\left(C\left(t\right)-\hat{C}\left(t\right)\right)-\mu \left(t\right)\left(N\left(t\right)-\hat{N}\left(t\right)\right) \end{array}\right\|\\ =\left\|\left(\begin{array}{c} -\left(\lambda \left(t\right)+\theta \left(t\right)\right),\lambda \left(t\right)\\ -\left(\nu \left(t\right)+\delta \left(t\right)\right),-\mu \left(t\right) \end{array}\right)\left(\begin{array}{c} C\left(t\right)-\hat{C}\left(t\right)\\ N\left(t\right)-\hat{N}\left(t\right) \end{array}\right)\right\|\\ \leq \left\|\left(\begin{array}{c} -\left(\lambda \left(t\right)+\theta \left(t\right)\right),\lambda \left(t\right)\\ -\left(\nu \left(t\right)+\delta \left(t\right)\right),-\mu \left(t\right) \end{array}\right)\right\|\cdot \left\|X-\hat{X}\right\|\leq \left\|A\left(t\right)\right\|\cdot \left\|X-\hat{X}\right\|, \end{array}$$

where

(13) $$\begin{array}{c} A\left(t\right)=\left(\begin{array}{c} -\left(\lambda \left(t\right)+\theta \left(t\right)\right),\lambda \left(t\right)\\ -\left(\nu \left(t\right)+\delta \left(t\right)\right),-\mu \left(t\right) \end{array}\right). \end{array}$$

It follows from the definition of random matrix and Hypothesis 2.1(ii) that we obtain

(14) $$\begin{array}{c} \left\|b\left(X,t\right)-b\left(\hat{X},t\right)\right\|\leq L\cdot \left\|X-\hat{X}\right\|, \end{array}$$

where

(15) $$\begin{array}{c} \left\|A\right\|\leq \underset{t\in \left[0,T\right]}{\max}{\left[E\left[{\left(\lambda \left(t\right)+\theta \left(t\right)\right)}^{2}+\lambda^{2}\left(t\right)+{\left(\nu \left(t\right)+\delta \left(t\right)\right)}^{2}+\mu^{2}\left(t\right)\right]\right]}^{1/2}\leq \sqrt{7}J≔L. \end{array}$$

By the similar way, we can prove that

(16) $$\begin{array}{c} \left\|B\left(X,t\right)-B\left(\hat{X},t\right)\right\|=\left\|\left(\begin{array}{c} g_{1}\left(t\right)\left(C\left(t\right)-\hat{C}\left(t\right)\right)\\ g_{2}\left(t\right)\left(C\left(t\right)-\hat{C}\left(t\right)\right) \end{array}\right)\right\|=\left\|\left(\begin{array}{c} g_{1}\left(t\right)\\ g_{2}\left(t\right) \end{array}\right)\cdot \left(X-\hat{X}\right)\right\|. \end{array}$$

It follows from the definition of random matrix and Hypothesis 2.1 (iii) that we obtain

(17) $$\begin{array}{c} \left|\left|B\left(X,t\right)-B\left(\hat{X},t\right)\right|\right|\leq K_{2}\left\|X-\hat{X}\right\|. \end{array}$$

This completes the check of the first hypothesis. Last but not least, it follows from Hypothesis 2.1 (iv) that

(18) $$\begin{array}{c} \left\|b\left(X,t\right)\right\|=\left\|\begin{array}{c} -\left(\lambda \left(t\right)+\theta \left(t\right)\right)C\left(t\right)+\lambda \left(t\right)N\left(t\right)\\ -\left(\nu \left(t\right)+\delta \left(t\right)\right)C\left(t\right)-\mu \left(t\right)N\left(t\right) \end{array}\right\|\\ =\left\|\left(\begin{array}{c} -\left(\lambda \left(t\right)+\theta \left(t\right)\right),\lambda \left(t\right)\\ -\left(\nu \left(t\right)+\delta \left(t\right)\right),-\mu \left(t\right) \end{array}\right)\left(\begin{array}{c} C\left(t\right)\\ N\left(t\right) \end{array}\right)\right\|\leq \left\|A\right\|\cdot \left\|X\right\|\leq L\left\|X\right\|\leq L\left(1+\left\|X\right\|\right). \end{array}$$

By the same way, we can obtain that

(19) $$\begin{array}{c} \left\|B\left(X,t\right)\right\|=\left\|\left(\begin{array}{c} g_{1}\left(t\right)\\ g_{2}\left(t\right) \end{array}\right)\right\|\leq K_{2}\leq L\leq L\left(1+\left\|X\right\|\right). \end{array}$$

This completes the check of the second hypothesis.

Therefore, the conclusion of Theorem 3.2 follows from Lemma 3.1. The proof is finished.

### 3.2. Mean-Square Asymptotical Stability

In this section, we investigate the mean-square uniformly asymptotic stability of the solution *φ*(*s*, *t*, *ω*)*X*_0_ of SDE (1). The pullback method is a powerful tool to the proof of uniformly asymptotic stability. To be precise, let us introduce some related definition [18].

**Definition 1 .**

The solution *φ*(*s*, *t*, *ω*)*X*_0_ of SDE (1) is said to be mean-square asymptotically stable if, for any given *ϵ* > 0, every other solution $\varphi \left(s,t,\omega \right){\hat{X}}_{0}$ of SDE (1) satisfies

(20) $$\begin{array}{c} \underset{t⟶+\infty }{\lim}\left\|\varphi \left(s,t,\omega \right)X_{0}-\varphi \left(s,t,\omega \right){\hat{X}}_{0}\right\|=0, \end{array}$$

for any bounded *ℱ*_*s*_-measurable bounded initial values *X*_0_ and ${\hat{X}}_{0}$ , respectively, where $\left\|X_{0}-{\hat{X}}_{0}\right\|<\varepsilon$ .

**Theorem 2 .**

Assume that for any initial values *X*_0_ and ${\hat{X}}_{0}\in L^{2}\left(\Omega ,\mathbb{P}\right)$ , the coefficients of SDE (1) satisfy Theorem 3.2, then the solution *φ*(*t* − *τ*, *t*, *θ*_−*τ*_ *ω*)*X*_0_ of SDE (1) is mean-square asymptotically stable.

**Proof**

First and foremost, let $\varphi \left(t-\tau ,t,\theta_{-\tau }\omega \right){\hat{X}}_{0}$ be another solution of SDE (1) and *ϵ* > 0 be an arbitrary constant. If $\left\|X_{0}-{\hat{X}}_{0}\right\|\leq \epsilon$ , it follows from (9) and the method which is used to estimate [10] that

(21) $$\begin{array}{c} E{\left|\varphi \left(t-\tau ,t,\theta_{-\tau }\omega \right)X_{0}-\varphi \left(t-\tau ,t,\theta_{-\tau }\omega \right){\hat{X}}_{0}\right|}^{2}\leq I_{1}+I_{2}+I_{3}, \end{array}$$

(22) $$\begin{array}{c} I_{1}=3E{\left|X_{0}-{\hat{X}}_{0}\right|}^{2}, \end{array}$$

(23) $$\begin{array}{c} I_{3}=3E{\left|\int_{t-\tau }^{t}B\left(\varphi \left(t-\tau ,t,\theta_{-\tau }\omega \right)X_{0},r\right)-B\left(\varphi \left(t-\tau ,t,\theta_{-\tau }\omega \right){\hat{X}}_{0},r\right)dW_{r}\right|}^{2}. \end{array}$$

It follows from the Cauchy–Schwarz inequality and the Lipschitz condition of the function *b* that we have

(24) $$\begin{array}{c} I_{2}\leq 3\tau \int_{t-\tau }^{t}E{\left|b\left(\varphi \left(t-\tau ,t,\theta_{-\tau }\omega \right)X_{0},r\right)-b\left(\varphi \left(t-\tau ,t,\theta_{-\tau }\omega \right){\hat{X}}_{0},r\right)\right|}^{2}dr\\ \leq 3\tau \cdot L^{2}\int_{t-\tau }^{t}E{\left|\varphi \left(t-\tau ,t,\theta_{-\tau }\omega \right)X_{0}-\varphi \left(t-\tau ,t,\theta_{-\tau }\omega \right){\hat{X}}_{0}\right|}^{2}dr. \end{array}$$

The Itô isometry and the global Lipschitz condition of the function *B* imply that

(25) $$\begin{array}{c} I_{3}\leq 3\int_{t-\tau }^{t}E{\left|b\left(\varphi \left(t-\tau ,t,\theta_{-\tau }\omega \right)X_{0},r\right)-B\left(\varphi \left(t-\tau ,t,\theta_{-\tau }\omega \right){\hat{X}}_{0},r\right)\right|}^{2}dr\\ \leq 3L^{2}\int_{t-\tau }^{t}E{\left|\varphi \left(t-\tau ,t,\theta_{-\tau }\omega \right)X_{0}-\varphi \left(t-\tau ,t,\theta_{-\tau }\omega \right){\hat{X}}_{0}\right|}^{2}dr. \end{array}$$

By the Gronwall inequality, there exists a number *M*_1_ such that

(26) $$\begin{array}{c} \left\|\varphi \left(t-\tau ,t,\theta_{-\tau }\omega \right)X_{0}-\varphi \left(t-\tau ,t,\theta_{-\tau }\omega \right){\hat{X}}_{0}\right\|\leq M_{1}, \end{array}$$

where

(27) $$\begin{array}{c} M_{1}=\sqrt{3}\left\|X_{0}-{\hat{X}}_{0}\right\|\sqrt{3\left(\tau +1\right)L^{2}\text{exp}\tau }. \end{array}$$

Therefore, by the fact that *M*_1_⟶0 as *τ*⟶−*∞*, we obtain that

(28) $$\begin{array}{c} \underset{t⟶+\infty }{\lim}\left\|\varphi \left(t-\tau ,t,\theta_{-\tau }\omega \right)X_{0}-\varphi \left(t-\tau ,t,\theta_{-\tau }\omega \right){\hat{X}}_{0}\right\|=0. \end{array}$$

Then, by Definition 3.1, it is mean-square asymptotically stable.

This completes the proof.

### 3.3. Mean-Square Convergence

The finite time interval [0, *t*] is divided into *N* subintervals with the length Δ*t*. The exact solution of SDE (1) in [0, *t*] has the form

(29) $$\begin{array}{c} X\left(t,\omega \right)=X_{0}+\int_{0}^{N\Delta t}b\left(X_{0},r\right)dr+\int_{0}^{N\Delta t}B\left(X_{0},r\right)dW_{r}. \end{array}$$

The Euler–Maruyama scheme is applied to SDE (6), and we have the following form

(30) $$\begin{array}{c} X_{k+1}=X_{k}+b\left(X_{k},t_{k}\right)\Delta t_{k}+B\left(X_{k},t_{k}\right)\Delta W_{k}, \end{array}$$

where *t*_*k*_=*k*Δ*t*, *k*=0,1,2,…, *N* and Δ*W*_*k*_=*W*_*k*+1_ − *W*_*k*_.

The Milstein scheme is applied to SDE (6), and we have the following form

(31) $$\begin{array}{c} X_{k+1}=X_{k}+b\left(X_{k},t_{k}\right)\Delta t_{k}+B\left(X_{k},t_{k}\right)\Delta W_{k}+\frac{1}{2}B\left(X_{k},t_{k}\right)\frac{\partial B\left(X_{k},t_{k}\right)}{\partial X}\left({\left(\Delta W_{k}\right)}^{2}-\Delta t_{k}\right). \end{array}$$

The following result shows that the numerical approximation *X*_*k*_ to the solution of SDE (1) is mean-square convergent to the exact solution of SDE (1) under some conditions.

**Theorem 3 .**

Assume that, for any initial value *X*_0_ ∈ *L* ^2^(*Ω*, *ℙ*), the coefficients of SDE (1) satisfy Theorem 3.3; then, the numerical approximation *X*_*k*_ to the solution of SDE (1) by Euler–Maruyama scheme and Milstein scheme is mean-square convergent, and the convergence order is 0.5.

**Proof**

We are interested in the mean square convergence to zero of the error

(32) $$\begin{array}{c} e_{k}=X_{k}-X\left(t_{k},\omega \right), \end{array}$$

where *X*(*t*_*k*_, *ω*) denotes the theoretical solution of SDE (1) at the time *t*_*k*_. From the expression of *X*(*t*, *ω*), we obtain

(33) $$\begin{array}{c} X\left(t_{k+1},\omega \right)=X\left(t_{k},\omega \right)+\int_{k\Delta t}^{\left(k+1\right)\Delta t}b\left(X\left(t_{k},\omega \right),r\right)dr+\int_{k\Delta t}^{\left(k+1\right)\Delta t}B\left(X\left(t_{k},\omega \right),t_{k}\right)dW_{r}. \end{array}$$

Then, it implies that

(34) $$\begin{array}{c} E{\left|X_{k+1}-X\left(t_{k+1},\omega \right)\right|}^{2}\leq I_{4}+I_{5}+I_{6}, \end{array}$$

where

(35) $$\begin{array}{c} I_{4}=3E{\left|X_{k}-X\left(t_{k},\omega \right)\right|}^{2}, \end{array}$$

(36) $$\begin{array}{c} I_{6}=3E{\left|B\left(X_{k},t_{k}\right)\Delta w_{k}-\int_{k\Delta t}^{\left(k+1\right)\Delta t}B\left(X\left(t_{k},\omega \right),r\right)dW_{r}\right|}^{2}. \end{array}$$

We notice from the Cauchy–Schwarz inequality and the global Lipschitz condition of function *b* that we can obtain

(37) $$\begin{array}{c} I_{5}\leq 3E{\left|\int_{k\Delta t}^{\left(k+1\right)\Delta t}b\left(X_{k},r\right)dr-\int_{k\Delta t}^{\left(k+1\right)\Delta t}b\left(X\left(t_{k},\omega \right),r\right)dr\right|}^{2}\\ \leq 3\Delta t_{k}L^{2}\int_{k\Delta t}^{\left(k+1\right)\Delta t}E{\left|X_{k}-X\left(t_{k},\omega \right)\right|}^{2}dr. \end{array}$$

Then, the Itô isometry and the global Lipschitz condition of the function *B* imply that

(38) $$\begin{array}{c} I_{6}\leq 3E{\left|\int_{k\Delta t}^{\left(k+1\right)\Delta t}B\left(X_{k},r\right)dW_{r}-\int_{k\Delta t}^{\left(k+1\right)\Delta t}B\left(X\left(t_{k},\omega \right),r\right)dW_{r}\right|}^{2}\\ \leq 3L^{2}\int_{k\Delta t}^{\left(k+1\right)\Delta t}E{\left|X_{k}-X\left(t_{k},\omega \right)\right|}^{2}dr. \end{array}$$

It follows from the Gronwall inequality that there exists a number *M*_2_ such that

(39) $$\begin{array}{c} E{\left|X_{k+1}-X\left(t_{k+1},\omega \right)\right|}^{2}\leq M_{2}, \end{array}$$

where

(40) $$\begin{array}{c} M_{2}=3E{\left|X_{k}-X\left(t_{k},\omega \right)\right|}^{2}\cdot 3\left(\Delta t_{k}+1\right)L^{2}\exp\;\;\Delta t_{k}. \end{array}$$

By the fact that *M*_2_ tends to zero as Δ*t*_*k*_⟶0, that is,

(41) $$\begin{array}{c} \underset{\Delta t_{k}⟶0}{\lim}M_{2}=0, \end{array}$$

we obtain

(42) $$\begin{array}{c} \underset{\Delta t_{k}⟶0}{\text{lim}}\left|\left|e_{k+1}\right|\right|=\underset{N⟶+\infty }{\text{lim}}\left\|X_{k+1}-X\left(t_{k+1},\omega \right)\right\|=0. \end{array}$$

Therefore, it is mean-square convergent, and the convergence order is 0.5. We have established the theorem.

## 4. Numerical Experiments

### 4.1. Experimental Preparation

Based on the sampling statistical data from Fujian Province, PR China, shown in Table 1, we consider the following stochastic differential equations of diabetes mellitus:

(43) $$\begin{array}{c} \left\{\begin{array}{c} dC\left(t\right)=\left(-0.03tC\left(t\right)+0.02tN\left(t\right)\right)dt+K_{3}\sin\;tdW_{t},\\ dN\left(t\right)=\left(0.05t-0.007tC\left(t\right)-0.002tN\left(t\right)\right)dt+K_{3}\cos\;tdW_{t}. \end{array}\right. \end{array}$$

That is,

(44) $$\begin{array}{c} \lambda \left(t\right)=0.02t,\\ \theta \left(t\right)=0.01t,\\ I\left(t\right)=0.05t,\\ \nu \left(t\right)+\delta \left(t\right)=0.007t,\\ \mu \left(t\right)=0.002t, \end{array}$$

(45) $$\begin{array}{c} g_{1}\left(t\right)=K_{3}\text{sin }t,\\ g_{2}\left(t\right)=K_{3}\text{cos }t, \end{array}$$

which are obtained by the fitting method in a one-dimensional space of real numbers. And, due to the randomness of sampling data and the periodic property of the considered noise, the sine and cosine functions are used to control the intensity of the added noise.

It follows from Theorem 3.2 that there exist solutions of SDE (43). As shown in [10], in order to obtain the Brownian trajectory, we can construct the positive time path and reflect it against point zero. We select the meshes as follows [14, 19]:

(46) $$\begin{array}{c} t=500,\\ \Delta t=0.01,\\ N+1=501. \end{array}$$

Brownian trajectories are generated by the following method:

(47) $$\begin{array}{c} W_{0}=0,\\ W_{\left(i+1\right)\Delta t}=W_{i\Delta t}+\psi_{i+1}, \end{array}$$

where

(48) $$\begin{array}{c} \psi_{i}=N\left(0,\sqrt{\Delta t}\right),\;i=1,2,\ldots ,N+1. \end{array}$$

Utilizing Theorem 3.2 and the one-step numerical scheme (EM scheme [19]) to solve SDE (43) with the initial value *X*_0_, we obtain

(49) $$\begin{array}{c} \left\{\begin{array}{c} C_{k+1}\left(t\right)=C_{k}\left(t\right)+\left(-0.03t_{k}C_{k}\left(t\right)+0.02t_{k}N_{k}\left(t\right)\right)\Delta t_{k}+K_{3}\sin\;t_{k}\Delta W_{k},\\ N_{k+1}\left(t\right)=N_{k}\left(t\right)+\left(0.05t_{k}-0.007t_{k}C_{k}\left(t\right)-0.002t_{k}N_{k}\left(t\right)\right)\Delta t_{k}+K_{3}\cos\;t_{k}\Delta W_{k}. \end{array}\right. \end{array}$$

Then, we obtain a numerically computed solution of SDE (43).

**Figure 1:**
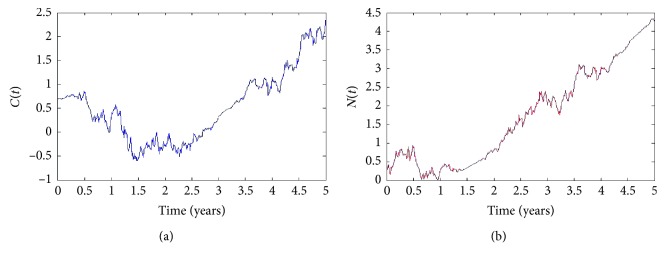
Numerical solutions *C*(*t*) and *N*(*t*) with corresponding different starting points *X*_0_=0.65 and *X*_0_=0.1 and *K*_3_=5.0.

And, we obtain the graphs for numerical approximations to the solutions in the time interval [0,500] as shown in Figure 1.

As we see, there exist random phenomena with different starting points *X*_0_=0.65 and *X*_0_=0.1 at time *t*=0. And, it shows the fact that the numerical results can match the reality very well, where the reality means the real statistic data of diabetes mellitus in Fujian Province, PR China.

### 4.2. Numerical Results

**Figure 2:**
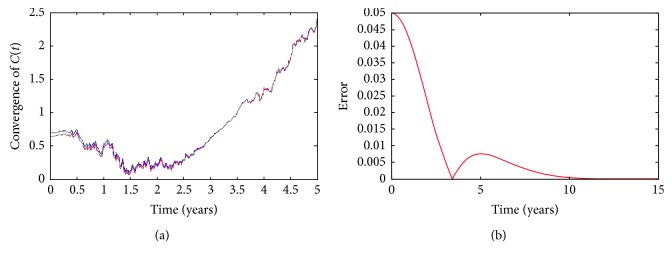
Convergence of the numerical solution *C*(*t*) with different starting points.

**Figure 3:**
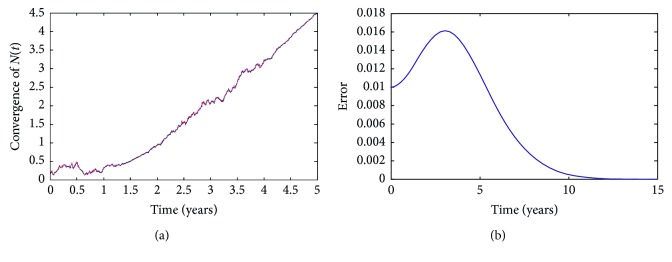
Convergence of the numerical solution *N*(*t*) with different starting points.

To begin with, in order to check the convergence of numerical approximations, we plot the curves from different starting points at the time *t*=0 in the same graph. In Figures 2 and 3, their starting points (*X*_0_, *Y*_0_) are (0.70, 0.10) and (0.65, 0.11), respectively. As time goes on, the trajectories tend to be more and more close and the difference of two numerical solutions becomes zero, too. This presents the fact that whatever starting points we select, two numerical solutions arrive at the same trajectory when time goes forward. That is to say, the solution of SDE (49) is a stochastic process which is different for every *ω* ∈ *Ω*. These confirm the fact that the numerical results are close to the epidemic disease tendency of diabetes mellitus in Fujian Province, PR China.

**Figure 4:**
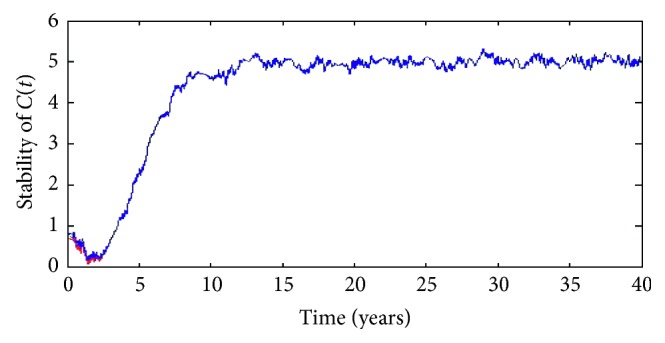
Stability of the numerical solution *C*(*t*) in 40 years.

**Figure 5:**
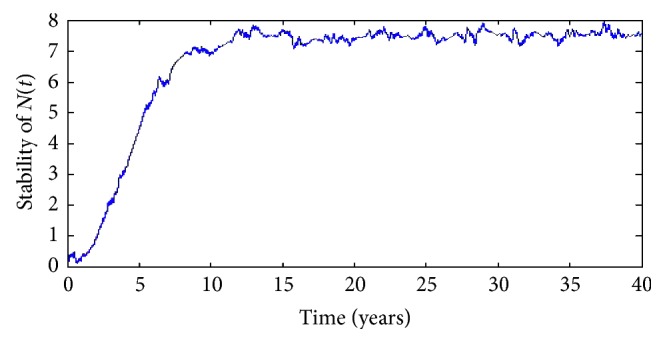
Stability of the numerical solution *N*(*t*) in 40 years.

Secondly, to check the stability of the true solution, we plot the curves from different starting points at time *t*=0 in the same graph in a long time, such as 40 years. As we see from Figures 4 and 5, the starting points (*X*_0_, *Y*_0_) are (0.70, 0.10), (0.65, 0.11), and (0.79, 0.09), respectively. As time progresses, the trajectories become close in a given small neighbourhood of one orbit. This also reflects the fact that if the starting points we choose are in a given region, as we move forward in time, numerical solutions arrive at the tolerant neighbourhood of one orbit which depend on different *ω* ∈ *Ω*. In other words, the solution of SDE (49) is not sensitive to the change of the initial value.

### 4.3. Control Policy and Its Practical Use

As we can see, the graph of *N*(*t*) shows that the total number of diabetes mellitus oscillates at the beginning and increases later. However, the graph of *C*(*t*) shows that the number of diabetes mellitus with complication decreases at first and then increases, too. Moreover, Figures 4 and 5 also present the fact that *N*(*t*) and *C*(*t*) both can exist random periodic phenomenon with the period of approximately 10 years. Therefore, efforts should be taken to move the situation out of the current endemic case. That is, we need to employ proper measure, such as nutrition supplement, gene therapy, physical activity and health education, and the number of incidence of diabetes mellitus will not exacerbate at least.

## 5. Conclusion

The main result of this article is the numerical simulation of stochastic diabetes mellitus model. The results show that the methods are effective and the numerical results can match the results of theoretical analysis and reality. Although some progresses are made, more simple and practical models and methods will be shown in our future work.

## Figures and Tables

**Table 1:** Sampling statistical data from Fujian Province, PR China, in five years (2012–2016).

Years	*λ*(*t*)	*θ*(*t*)	*I*(*t*)	*ν*(*t*)+*δ*(*t*)	*μ*(*t*)
2012	0.0211	0.0089	0.0573	0.00526	0.00191
2013	0.0401	0.0101	0.1010	0.00611	0.00081
2014	0.0612	0.0293	0.1536	0.00650	0.00118
2015	0.0812	0.0387	0.2006	0.00706	0.00157
2016	0.0991	0.0591	0.2436	0.00664	0.00198

## Data Availability

The data used to support the findings of this study is real and reliable.
